# Evaluation of a genicular axial pattern flap to repair large cutaneous tibial defects in two dogs

**DOI:** 10.1186/s12917-019-1900-2

**Published:** 2019-05-22

**Authors:** Ciprian Ober, Joshua Milgram, William McCartney, Marian Taulescu, Cosmin Pestean, Iulia Melega, Liviu Oana

**Affiliations:** 1 University of Agricultural Sciences and Veterinary Medicine, Calea Manastur 3-5, Cluj-Napoca, Romania; 2 The Koret School of Veterinary Medicine, Yehoshua Hankin St 21, Rehovot, Israel; 3 NOAH, 38 Warrenhouse Road, Baldoyle, Dublin 13, Ireland

**Keywords:** Axial pattern flap, Dog, Genicular artery

## Abstract

**Background:**

Clinical outcome after cutaneous reconstruction using genicular artery flaps has not been reported. Major cutaneous defects of the pelvic limb between the stifle and hock are frequent in dogs and closure is difficult due to lack of available skin from immediately adjacent areas.

**Case presentation:**

Here we report the first two clinical cases successfully managed by genicular axial pattern flap closure. A 2-year-old 38 kg (83.77-lb) intact male Labrador Retriever and a 14-year-old 42 kg (92.59-lb) spayed mixed breed female dog were admitted for the management of large skin defects in the lateral tibiotarsal joint. One defect was the result of a fibrosarcoma removal in the Labrador dog and the other defect was a chronic large wound caused by a car accident in the mixed breed female dog. Both defects were reconstructed by using genicular flaps. The bed of the wound in mixed breed dog was surgically debrided and underwent open wound management until a proper granulation tissue bed was formed before reconstruction. The skin defect in the Labrador dog was covered immediately after tumor removal. After surgery both dogs were bearing weight on the limbs normally. Small area of dehiscence occurred in both dogs 2 weeks after surgery. At follow-up examination one month after surgery, the surgical wound of the Labrador retriever still had a small area of dehiscence. Two months after surgery, the wound of the mixed breed dog was completely healed, covered with hair and no lameness was observed.

**Conclusion:**

Findings suggested that genicular axial pattern flap is a good option for reconstruction of large cutaneous defects of the lateral aspects of the tibia in dogs.

## Background

Closure of major cutaneous defects of the pelvic limb between the stifle and hock is difficult due to lack of available skin from immediately adjacent areas [1, 2]. Depending on the rear limb length of the patient, an axial pattern flap based on the caudal superficial epigastric vessels [3] may not be of sufficient length to cover a skin defect at a level below the proximal tibia.

Axial pattern flaps incorporate a direct cutaneous artery and vein with terminal branches for blood flow supply and drainage of the subdermal plexus [4]. The genicular axial pattern flap is based on the genicular branches of the saphenous artery and medial saphenous vein [4]. The genicular artery extends cranially over the medial aspect of the stifle and terminates over its craniolateral surface. This axial pattern flap is a local skin flap which may be developed and transferred in one stage to cover cutaneous defects of the proximal two thirds of the tibia [5]. The flap provides immediate full thickness skin coverage and offers several advantages over other techniques currently used to treat cutaneous defects in this area [5]. Compared with random or subdermal plexus flaps, incorporation of direct cutaneous vessels allows for a larger flap with more consistent survival [1].

Advantages of axial pattern flaps over other methods of wound closure include the ability to close a large defect without tension, early closure without extended open wound management, coverage of areas with less than optimal wound healing conditions, and excellent flap survival rates [6, 7]. The overall survival rate of axial pattern flaps is 87 to 100% [7–13]. Although the tips of the flaps are prone to necrosis, mean survival of axial pattern flaps is at least 50% greater than of subdermal plexus flaps [14, 15].

The genicular axial pattern flap has been described for repair of defects from stifle to tibiotarsal joint on the lateral or medial aspect of the hindlimb [4, 5], but, to the authors’ knowledge, clinical outcome following reconstruction of cutaneous defects by using genicular artery flaps has not been reported in the literature.

## Case presentation

**Fig. 1:**
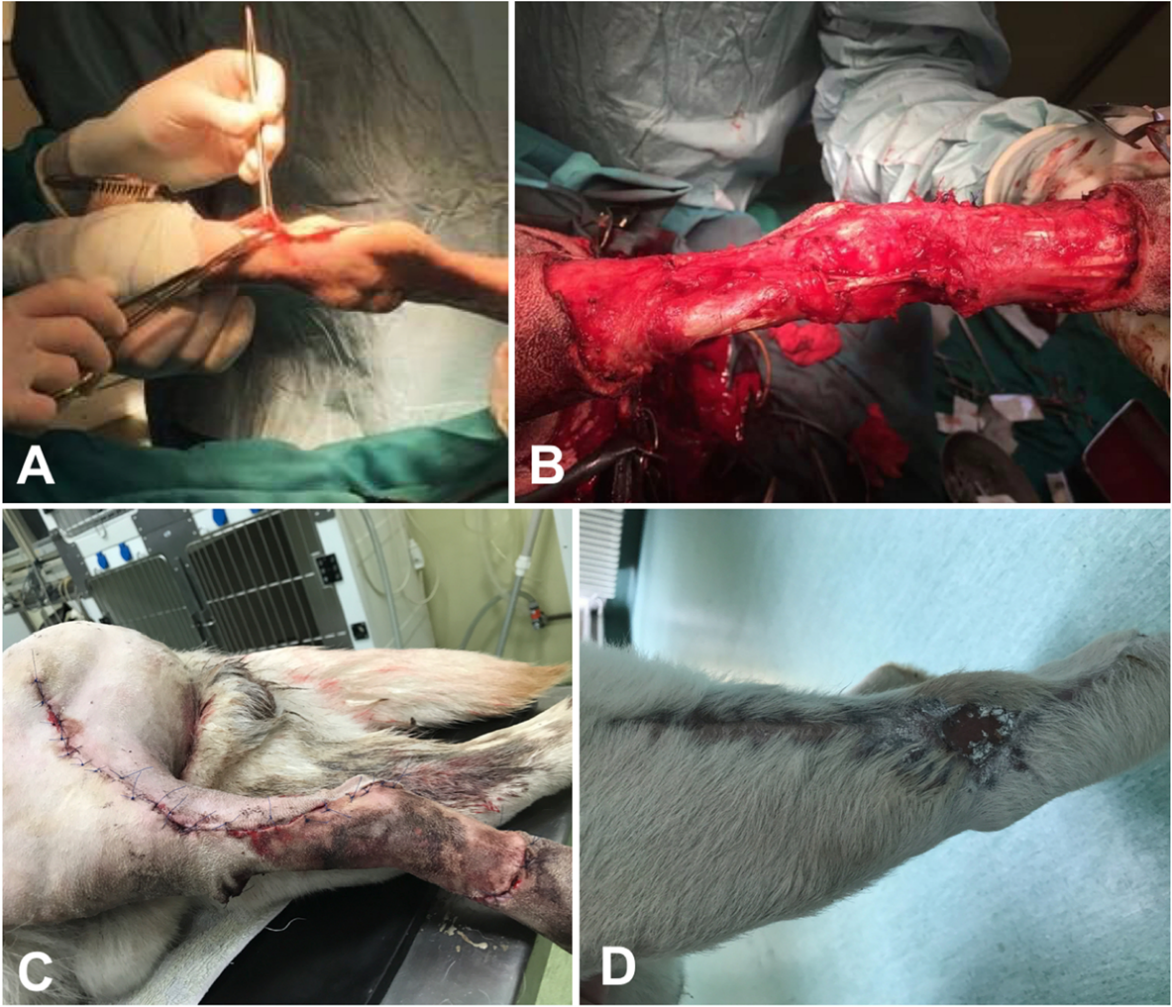
Distal tibial fibrosarcoma (**a**) and the defect resulted after wide excision (**b**) in a 2-year-old intact male Labrador. **c** Immediate postoperative aspect of the flap and aspect after 1 month postoperatively (**d**); notice the dehisced area with healing by second intention

A 2-year-old 38 kg (83.77-lb) intact male Labrador and a 14-year-old 42 kg (92.59-lb) neutered mixed breed female dog were referred to the Department of Surgery the Faculty of Veterinary Medicine at the University of Agricultural Sciences and Veterinary Medicine Cluj-Napoca, Romania. On physical examination, the Labrador dog had a dense, 3X4 cm well-defined subdermal mobile swelling on the craniolateral aspect of the tibia (Fig. 1a).

General condition of the dog was excellent and no lameness was observed on the affected limb. The dog had been evaluated by the referring veterinarian who also performed a biopsy. Histologic evaluation established a diagnosis of intermediate (grade 2) fibrosarcoma. Thoracic radiography and abdominal ultrasound revealed no metastatic foci. CT scan analysis was not available. No evidence of regional lymph node involvement was noticed. All results of CBC and serum biochemical analysis were within reference limits. A 3 cm surgical excision was performed, because histologic examination of tissue margins indicating complete tumor resection is predictive of nonrecurrence, and unaffected tissue margins of 2 to 3 cm in all planes are typically recommended to achieve this goal [16] (Fig. 1b).

Cefazolin (22 mg/kg [10 mg/lb], IV) was administered 30 min prior to surgery, and the patient was premedicated with butorphanol (0.3 mg/kg [0.14 mg/lb], IM) and midazolam (0.3 mg/kg, IM). Anesthesia was induced with propofol (3 mg/kg [1.4 mg/lb], IV) and maintained with isoflurane in oxygen following endotracheal intubation. The patient was placed in lateral recumbency, and a hanging leg preparation was performed. The tumor was removed with 3 cm margins (Fig. 1b).

**Fig. 2:**
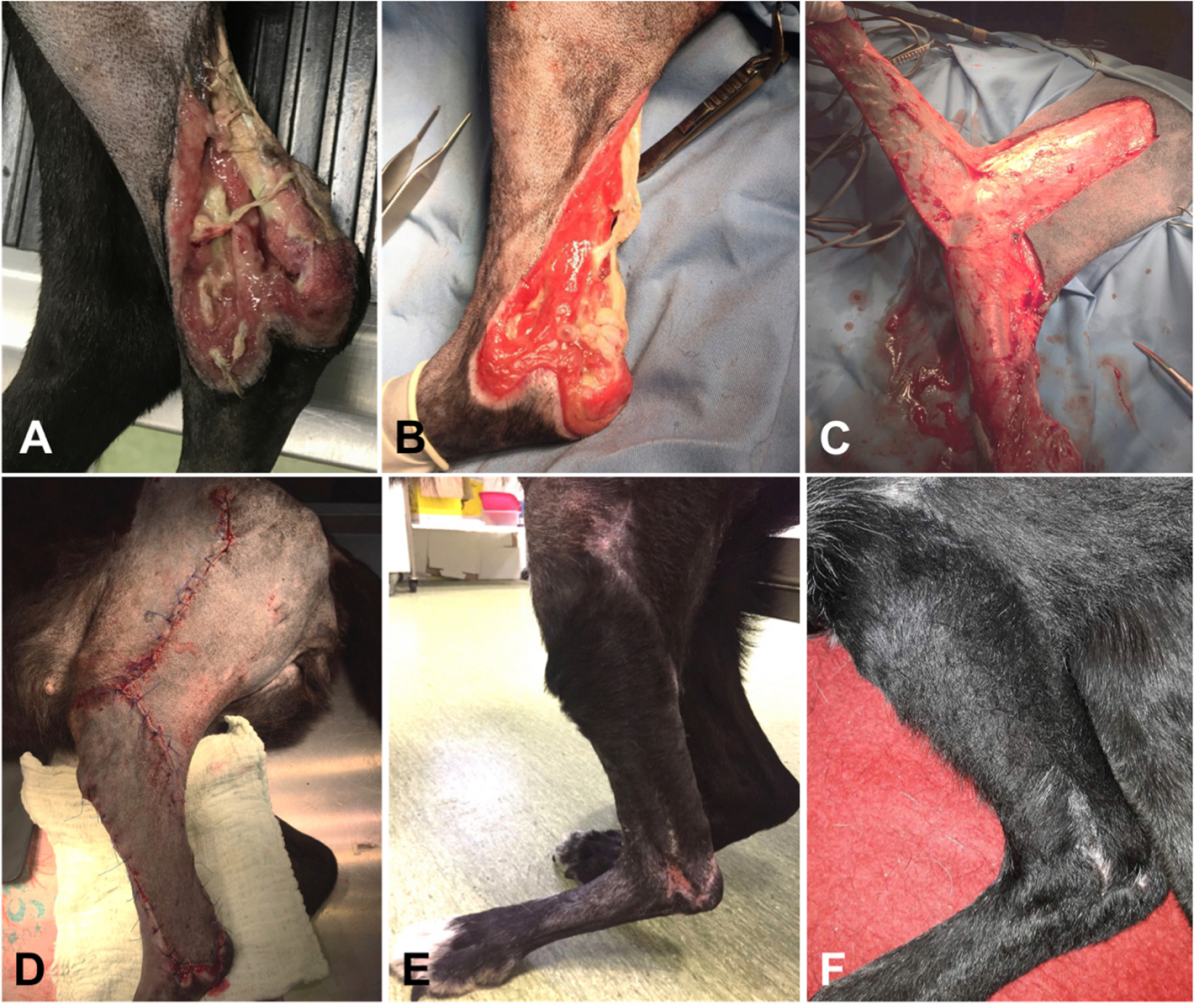
Large open wound at the level of left lateral distal tibia (**a**) in a 14-year-old spayed female mixed breed; inflammation, edema, and necrosis of periwound skin are present. **b** Recipient bed prepared for flap closure. **c** Elevation of the flap. **d** Closure of the donor bed and immediate postoperative appearance of the genicular axial pattern flap. **e** Small dehisced wound several days after breakdown at the tip of the closure. **f** Complete healing and hair growth after 2 months postoperative

The 14-year-old 42 kg (92.59-lb) neutered mixed breed female dog was referred because of a chronic and 14X6 cm extensive skin defect on the lateral aspect of the distal tibia and tarsus (Fig. 2a). The dog was found on the street; probably a road accident was the main cause of cutaneous blunt trauma. No orthopedic damage was present. Neural motor function of the limb was adequate. General condition of the dog was very good, and score 2 lameness was present on the affected limb. CBC and serum biochemistry values were all within reported reference ranges at the time of surgery. The wound was surgically debrided and managed open with foam dressings containing dextrans, alginate and silver coating until a recipient bed of granulation tissue was achieved (Fig. 2b). Several millimeters of skin bordering the recipient bed were removed with a scalpel blade until a proper granulation bad was formed. Wound bed tissue samples were obtained for culture and sensitivity testing.

Cefazolin (22 mg/kg [10 mg/lb], IV) was administered 30 min prior surgery, and the anesthesia was performed in a similar way to the previous case. The patient was placed in lateral recumbency, and a hanging leg preparation was performed. The proposed incision lines and measurements of the flap length and width were done in a similar way to the previous case.

The genicular axial pattern flaps were marked with a sterile pen and the flaps were then developed on the lateral aspect of the limb. The base of the flap was located 1 cm proximal to the patella and 1.5 cm distal to the tibial tuberosity on the lateral aspect of the limb according to the recommendations [1] (Fig. 2c). The incisions converged slightly so that the base was 2 cm wider than the tip [1]. Flaps were elevated in the loose areolar fascial plane below the skin according to recommendations [1]. Flap dimensions varied in length and width between the two dogs. The flaps were meticulously undermined, rotated distally to cover the cutaneous defects and sutured to the recipient site similar for both cases, using simple interrupted subcutaneous 3–0 monofilament polydioxanone and 3–0 monofilament nylon in interrupted pattern for the skin (Figs. 1c, 2d). The dogs recovered from anesthesia uneventfully. They were confined in cages having Elisabethan collars and the wounds were inspected every day for the presence of dehiscence and necrosis. Treatment with tramadol (3 mg/kg PO every 12 h, 5 days), carprofen (3 mg PO once daily, 5 days) and amoxicillin–clavulanic acid (18 mg/kg PO twice daily, 7 days) was established for both dogs.

Assessment of flaps viability was made 10 days, 4 and 8 weeks postoperative and was based on skin color and hair growth. A small dehiscence occurred in both dogs in the first two weeks postoperative (1X1 cm in the Labrador and 0,5X0,5 in the mixed breed dog). Due to the small size of the dehisced area it was decided to let the area heal by secondary intention for both cases (Fig. 2e). A small 1.5X1.5 cm skin defect remained in the Labrador (Fig. 1d) one month after surgery, but the owner was very satisfied with the outcome. At follow-up examination performed at 2 months, the surgical area in mixed breed dog was completely healed, covered by hair (Fig. 2f) and no lameness was observed.

## Discussion and conclusion

Reported complications in experimental cases included dehiscence of the donor site and necrosis of the flap. Mean flap survival was 89% in dogs that underwent genicular axial pattern flap elevation and rotation to cover a defect over lateral tibia [5]. The degree of flap necrosis ranged from 10 to 33% and was thought, in part, to result from variability in number and location of the genicular branches of the saphenous vessels [5]. Necrosis may be reduced with meticulous surgical technique, use of shorter flaps, and prevention of postoperative trauma. The genicular artery is not a large vessel; therefore, flaps should be kept as short as possible to avoid necrosis [1]. Incisional dehiscence was a postoperative complication in both cases and the dehiscence occurred at the tip of the flaps in the dogs. We assume that small dehiscences of the recipient site resulted because of long flaps were used to cover large defects combined with increased tension along the incision line and pressure exerted to the flap by bony prominence in the Labrador. Dehiscence may have been minimized by a better placement of tension sutures or by altering the length or width of the flap. However, both defects healed by secondary intention healing and did not require any further surgical intervention. No hair was present at the level of the healed defect at the last follow up in Labrador, but the cosmesis was satisfactory. Complete hair growth and no dehiscence was observed at the last follow-up in mixed breed dog. Although in some patients cosmesis may be less acceptable because of variations in coat length and color, the owners of the dogs in the present study were very satisfied with the outcome.

Although many cutaneous wounds of limbs heal by contraction and epithelialization, the process may be slow or incomplete, and the healed wound may have a fragile epithelial surface [5]. Second intention healing may lead to contracture, excessive scarring, and compromise of the venous and lymphatic return distal to the site of injury [2, 3, 17, 18]. Thus, axial pattern flaps applied to skin defects on limbs may bypass problems associated with second intention healing [5]. At the last follow up, no effect on venous and lymphatic return was observed in the Labrador that had a small dehiscence left to heal by second intention.

Surgery alone is curative if the tumor can be removed completely [16]. Open wound management after wide excision of distal extremity grade 1 and 2 soft tissue sarcoma resulted in a median disease-free survival of more than 980 days in 31 dogs [19]. Complications included intermittent bleeding because of the relatively fragile skin after epithelization in 16% and wound contracture concerning the carpal area in 10% of the cases. Faster healing and better skin quality can be achieved using a skin graft technique for wound closure [1]. Other alternatives for closure are direct full-thickness meshed skin grafts. In a larger retrospective study of 32 dogs success rate in dogs was only 32% [20]. Unlike free grafts, axial pattern flaps have an inherent blood supply and do not require a vascular bed for their initial survival [5]. The flaps are very durable, whereas durability of free grafts will vary according to graft thickness and the technique used [5]. Free grafts require immobilization of the affected limb [2, 3], whereas axial pattern flaps do not. Based on these previous findings, we considered for our cases an axial pattern flap with genicular artery as a better option.

This study shows that genicular flaps provided a full thickness skin coverage in two dogs with cutaneous defects in the tibiotarsal region.

## Authors’ information

CO is Resident of the European College of Veterinary Surgeons and Senior Lecturer in Small Animal Surgery at University of Agricultural Sciences and Veterinary Medicine, Faculty of Veterinary Medicine, Cluj-Napoca, Romania.

JM is Diplomate of the European College of Veterinary Surgeons and Senior Lecturer in Small Animal Surgery at The Koret School of Veterinary Medicine, Hebrew University of Jerusalem, Israel.

WMC is Diplomate of the European College of Veterinary Surgeons and Principal Surgeon at North Dublin Orthopaedic Animal Hospital, Dublin, Ireland.

MT is Diplomate of European College of Veterinary Pathologists and Associate Professor at University of Agricultural Sciences and Veterinary Medicine, Faculty of Veterinary Medicine, Cluj-Napoca, Romania.

CP is Associate Professor in Department of Anesthesia and Intensive Care at University of Agricultural Sciences and Veterinary Medicine, Faculty of Veterinary Medicine, Cluj-Napoca, Romania.

IM is PhD Student at Department of Anesthesia and Intensive Care at University of Agricultural Sciences and Veterinary Medicine, Faculty of Veterinary Medicine, Cluj-Napoca, Romania.

LO is Full Professor at Department of Small Animal Surgery at University of Agricultural Sciences and Veterinary Medicine, Faculty of Veterinary Medicine, Cluj-Napoca, Romania.
